# Body Weight at Birth: The Only Risk Factor Associated with Contralateral Clavicular Fracture in Patients with Congenital Muscular Torticollis

**DOI:** 10.1038/s41598-019-50370-2

**Published:** 2019-09-24

**Authors:** Zeeihn Lee, Joo Young Cho, Byung Joo Lee, Jong Min Kim, Donghwi Park

**Affiliations:** Department of Rehabilitation Medicine, Daegu Fatima Hospital, Daegu, South Korea

**Keywords:** Paediatric research, Risk factors

## Abstract

To date and to the best of our knowledge, there have been limited studies on the risk factor of clavicle fracture combined with congenital muscular torticollis (CMT), despite it being the most common fracture in newborns. So, the aim of this study was to investigate the risk factors associated with clavicular fracture combined with CMT, and its effect on prognosis. In this study, a total of 134 infants with CMT were included. The risk factors associated with clavicular fracture combined with CMT were analyzed. To analyze the correlation between the clinical parameters and the clavicular fracture in patients with CMT, demographic data, such as body weight at birth, maternal age, gender, gestational age, delivery method, sternocleidomastoid (SCM) thickness of ipsilateral side, its ratio between the ipsilateral and contralateral side, and the first visitation date after birth were evaluated. In the results of this study, the clavicular fracture was found in 15 of 134 patients with CMT (19%). In multivariate logistic analysis, the body weight at birth was the only significant parameter for predicting clavicular fracture in patients with CMT (p-value < 0.05). However, there was no significant difference of treatment duration between CMT infants with or without clavicular fracture. In infants with CMT, the area under the ROC curve of the body weight at birth for predicting clavicular fracture was 0.659 (95% CI, 0.564–0.745.; p < 0.05). The optimal cut-off value obtained from the maximum Youden index J was 3470 g (sensitivity: 57.14%, specificity: 75.76%), and the odd ratio of clavicular fracture in patients with CMT increased by 1.244 times for every 100 g of body weight at birth. In conclusion, birth weight appears to be a clinical predictor of clavicular fracture in infants with CMT. More studies and discussions are needed on whether any screening should be recommended for detecting the concurrent clavicular fracture in subjects with CMT.

## Introduction

Congenital muscular torticollis (CMT) is one of the most common musculoskeletal problems in children, and the overall incidence rate can be as high as 1:250 live births^1,2^. The reported incidence rate varied from 0.3 to 16.0%^1–5^. CMT is a group of clinical presentations caused by the shortening of the unilateral sternocleidomastoid muscle (SCM), which begins during the prenatal or perinatal period^6,7^. CMT can usually be subdivided into two phenotypes^2^; (1) the sternocleidomastoid tumor (SMT) type, which consists of torticollis with a palpable tumor (fibromatosis colli), and (2) the muscular torticollis, which consists of torticollis with a tightness of SCM without a palpable mass. The former is a mass that may be tender to palpation and decreases in size within the first year after birth. And postural torticollis (PT) may be classified as one of subtypes of CMT, although there are differences between studies^2,3^.

Congenital muscular torticollis (CMT) is known to occur concurrently with other conditions, including brachial plexus injury (BPI), developmental dysplasia of the hip (DDH), or clavicular fracture^8–11^. In previous study, the incidence of perinatal torticollis occurring concurrently with neonatal BPI was 43%^9^. And the incidence of perinatal torticollis occurring concurrently with neonatal BPI ranged from 2% to 19%^11^. To date and to the best of our knowledge, there have been limited studies on the risk factor of clavicle fracture combined with CMT, despite it being the most common fracture in newborns^12^. Although one previous study showed that clavicular fracture tends to develop on the contralateral side of CMT, the risk factors of clavicular fracture in patients with CMT have not been fully evaluated^6^. Therefore, the aim of this study was to investigate the risk factors associated with clavicular fracture in infants with CMT and its effect on prognosis.

## Material and Method

### Ethics statement

This study was approved by the Institutional Review Board of Daegu Fatima Hospital. Declaration of Helsinki protocols are being followed, and informed consent was obtained from a parent and/or legal guardian.

### Patients

Between January 2016 and June 2018, subjects who visited our rehabilitation outpatient clinic due to abnormal posture of the head and neck were included. The medical records along with radiological findings were reviewed. Among them, subjects who met the following criteria were excluded: (1) subjects with no specific finding on ultrasonography; (2) subjects who did not undergo plain radiography of the cervical spine and/or clavicles.

CMT was diagnosed when subjects met the following criteria: (1) thickness of the involved SCM ≥ 2 mm greater than that of the contralateral side, along with increased echogenicity on ultrasonography^13^; and (2) subjects who showed shortening of the unilateral SCM since childhood, ending up with a limitation of passive range of rotation of the chin toward ipsilateral shoulder and/or limitation of passive range of lateral flexion toward contralateral shoulder. Plain radiographs (X-ray) of the cervical spines and/or clavicles were evaluated, using the reference set to any clavicular fracture and any structural abnormalities on cervical spine. Clavicular fracture was diagnosed when the fracture lines and/or callus were detectable by a naked eye on the clavicle on antero-posterior plain radiographs of the cervical spines and/or clavicles^6^ and confirmed by radiologists who were specialized at musculoskeletal disease. Therefore, we retrospectively reviewed the medical records of 134 patients with congenital muscular torticollis in the rehabilitation unit.

### Parameters associated with clavicular fracture in CMT patients

Demographic data, such as body weight at birth, maternal age, gender, gestational age, delivery method, SCM thickness of ipsilateral side, and its ratio between the ipsilateral and contralateral side, treatment duration, in addition to the first visitation date after birth, were collected by reviewing the medical records.

### Statistical analysis

To find the difference of the demographic data between CMT patients with and without clavicular fracture, an independent T-test, Fisher’s exact test, or chi-square test were performed. In addition, to analyze the correlation between the clinical parameters and the clavicular fracture in patients with CMT, multivariate logistic analysis through forward stepwise selection was then performed. To evaluate the accuracy of predictive factors for clavicular fracture in CMT patients, we performed a receiver operating characteristic (ROC) analysis in each group. All statistical analyses were conducted using MedCalc and SPSS version. 22.0 (IBM, Armonk, NY, USA).

## Results

### Demographics and concurrence rate of CMT and clavicular fracture

**Figure 1:**
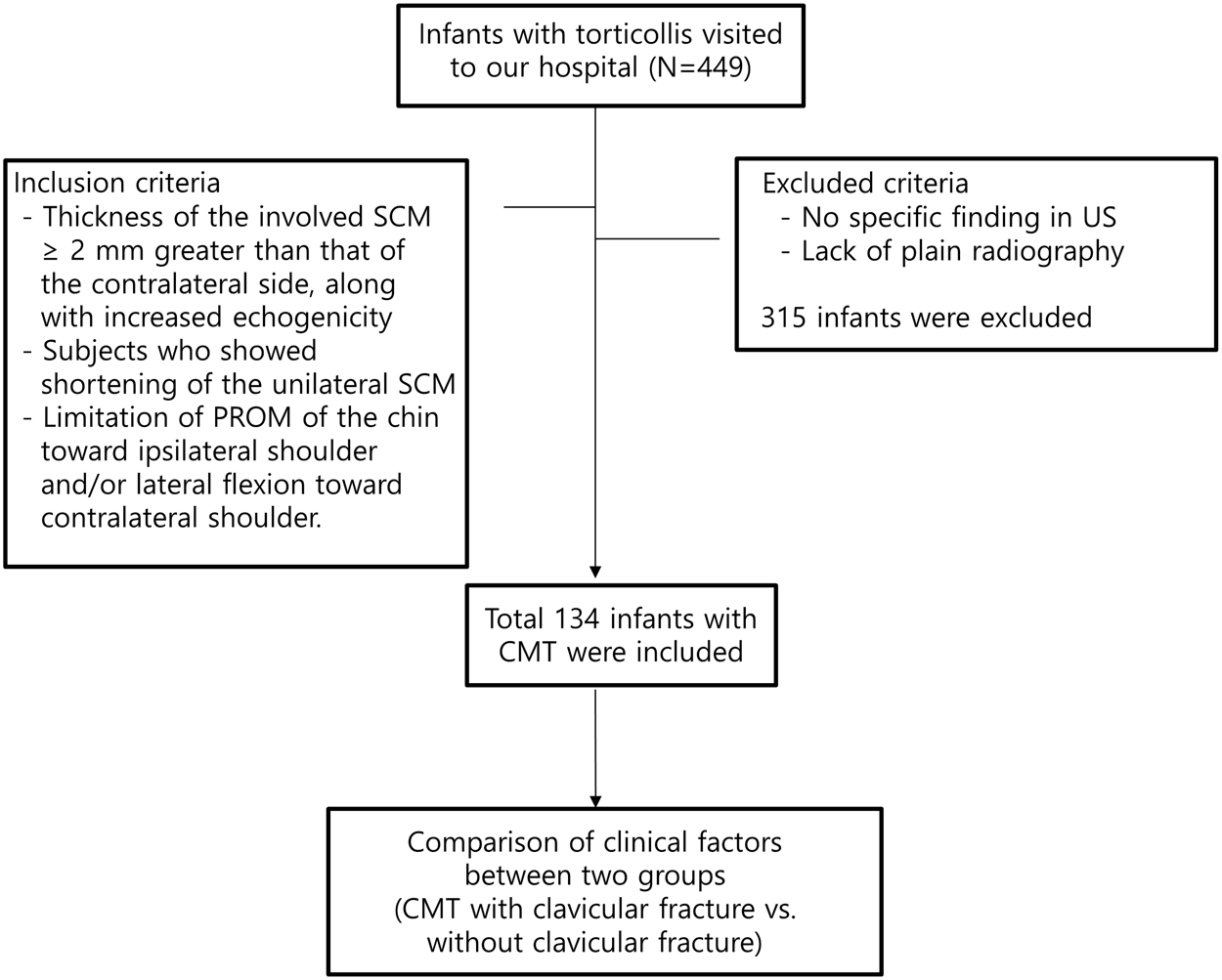
Flow chart of this study.

Between January 2016 and June 2018, 449 patients visited our rehabilitation outpatient clinic due to abnormal posture of the head and neck. The medical records along with radiological findings were reviewed. Among them, 315 subjects who met the exclusion criteria were excluded (Fig. 1). Finally, a total 134 infants with CMT were included in this study. Moreover, the clavicular fracture was found in 15 of 134 infants, with the concurrent rate being 11.19% (Table 1).

**Table 1:** Comparison of the patients with and without clavicle fracture in infants with congenital muscular torticollis.

Characteristic	Congenital muscular torticollis		
	Clavicle fracture (−)	Clavicle fracture (+)	p-value
Number	119	15	
Body weight at birth	3226.77 ± 380.871	3442.86 ± 362.628	0.048*
Gestational age	273.98 ± 7.56	274.21 ± 6.411	0.781
First visit at clinic (days)	46.93 ± 60.80	31.20 ± 26.61	0.086
Time interval between birth and making the diagnosis of CMT (days)	66.95 ± 70.65	43.40 ± 27.45	0.205
Male: Female	70:49	10:5	0.912
NVD: C-sec	67:39	14:0	0.005*
Number of breech	18	0	0.221
SCM thickness (ipsilateral)	1.117 ± 0.232	1.1844 ± 0.266	0.323
SCM thickness (contralateral)	0.704 ± 0.095	0.722 ± 0.717	0.500
SCM thickness ratio (ipsilateral/contralateral)	1.616 ± 0.409	1.663 ± 0.426	0.702
Treatment duration (days)	112.93±85.48	113.36±47.51	0.262

Values: mean ± standard deviation. *p < 0.05 compared between two groups, NVD; normal virginal delivery, C-sec; Caesarean section, SCM; Sternocleidomastoid.

### Contralateral involvement of CMT and clavicular fracture

CMT and clavicular fracture occurred on the opposite side of each other in 13 out of 15 subjects (86.7%; Table 2). Table 2 is the contingency table between the side of CMT and the side of clavicular fracture. In chi-square analysis, discordance of side between CMT and clavicular fracture was significant (P = 0.004).

**Table 2:** Contingency table between the side of clavicular fracture and the location of congenital muscular torticollis.

Side of CMT	Location of clavicular fracture		P-value
	Left	Right	
Right	4	0	0.004^*^
Left	2	9	

### Risk factor of clavicular fracture in infants with CMT

**Figure 2:**
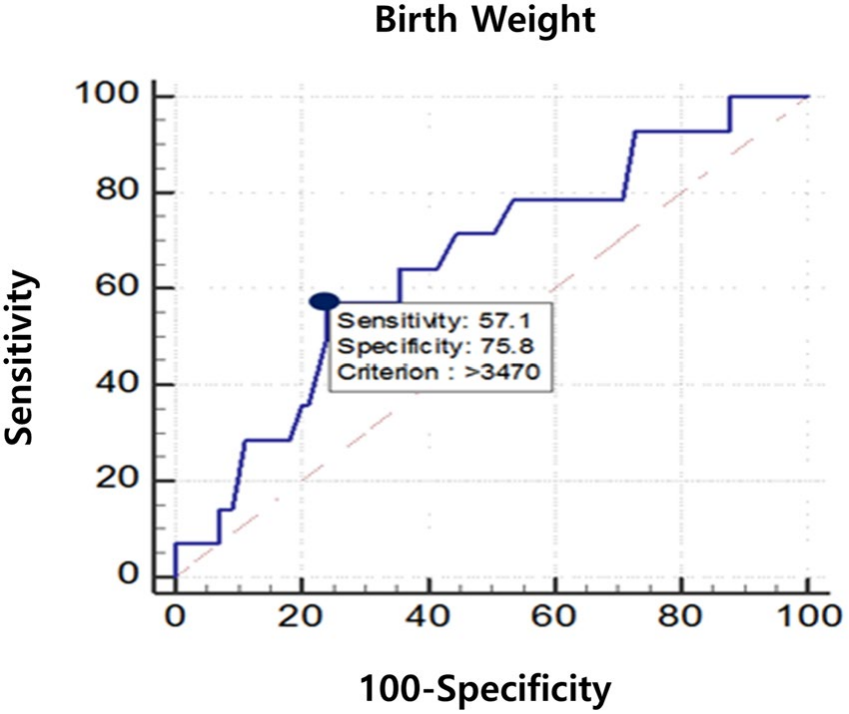
In patients with CMT, the area under the ROC curve of the body weight at birth for predicting clavicular fracture was 0.659 (95% CI, 0.564–0.745.; p < 0.05). The optimal cut-off value obtained from the maximum Youden index J was 3470 g (sensitivity: 57.14%, specificity: 75.76%), and the odd ratio of clavicular fracture in patients with CMT increased by 1.244 times at every 100 g of body weight at birth.

In a comparison of the demographic data between CMT infants with or without clavicular fracture, there was a significant difference in delivery mode (p-value < 0.05) (Table 1). There was a significant difference in the birth weight between CMT infants with or without clavicular fracture (p-value < 0.05) (Table 1). However, there was no significant difference in the maternal age, gestational age, SCM thickness ratio, first visitation to the clinic, and gender. In a multivariate logistic analysis, the birth weight was the only significant parameter for predicting clavicular fracture in CMT infants (p-value < 0.05) (Table 1). In infants with CMT, the area under the ROC curve of the birth weight for predicting clavicular fracture was 0.659 (95% CI, 0.564–0.745.; p < 0.05) (Table 3). The optimal cut-off value obtained from the maximum Youden index J was 3470 g (sensitivity: 57.14%, specificity: 75.76%), and the odd ratio of clavicular fracture in CMT infants increased by 1.244 times for every 100 g of birth weight (Fig. 2).

**Table 3:** Multivariate logistic regression analysis associated with clavicular fracture in patients with congenital muscular torticollis.

Parameter	Beta coefficient	Standard error	OR (95% CI)	p-value
Body weight at birth	0.219	0.0785	0.659 (0.564-0.745)	0.0431*

### Clinical parameters correlated with treatment duration

There was no significant difference of treatment duration between CMT infants with or without clavicular fracture. (Table 1) In a multivariate regression analysis, the SCM thickness ratio was the only significant parameter for predicting treatment duration in infants with CMT (p-value < 0.05) (Table 4).

**Table 4:** Multivariate regression analysis associated with treatment duration in patients with congenital muscular torticollis.

Parameter	Unstandardized coefficients		standardized coefficients		p-value	95% CI
	Beta coefficient	Standard error	Beta	t		
SCM thickness ratio (ipsilateral/contralateral)	47.12	17.45	0.272	2.701	0.008*	12.46–81.77

PT; physical therapy, treatment duration; the time between the initial treatment and the achievement of complete passive cervical rotational range of motion.

## Discussion

The causes of CMT remain contentious to date^2^. Multiple theories exist, including intrauterine crowding^8,14^ or fibrosis from peripartum bleeding, vascular phenomenon, primary myopathy of the SCM, and compartment syndrome^15,16^. A difficult birth history has been reported in 30–60% of patients with CMT^17,18^. In a study of 996 patients with CMT, Yim *et al*.^6^ suggested a hypothesis that CMT may likely develop during vaginal delivery. For an effective expulsion of the baby’s head during vaginal delivery, the antero-posterior axis of baby’s head needs to be parallel with the antero-posterior axis of the mother’s pelvis, by simultaneous internal rotation of both the head and trunk of the baby, in addition to neck flexion^6^. Moreover, external rotation of the shoulder occurs so that the right-left axis of the baby’s shoulders becomes parallel to the antero-posterior axes of the mother’s pelvis for expulsion of the baby’s shoulder^6^. However, Yim *et al*.^6^ suggested that an isolated internal rotation of the baby’s head that occurs during head expulsion, instead of simultaneous internal rotation of both the head and shoulder, can cause overstretching and damages to SCM. Moreover, they also suggested that a downward traction of the assistive maneuver, which may facilitate delayed delivery of the baby’s shoulder, may cause fracture of the clavicle (contralateral side of damaged SCM), which is in the anterior shoulder. As aforementioned, clavicular fracture is significantly correlated with vaginal delivery and body weight at birth. The suggestions made by Yim *et al*. and our findings support that clavicular fractures in patients with CMT are associated with difficult vaginal delivery. Interestingly, however, there was no significant difference of treatment duration between CMT infants with or without clavicular fracture. In this study, the only risk factor correlated with treatment duration was SCM thickness ratio.

Although other theories aside from difficult vaginal delivery, such as vascular phenomenon, primary myopathy of the SCM, compartment syndrome, hereditary hypothesis, and infection, have also been proposed, the pathogenesis of CMT remains uncertain. Injury to the SCM muscle can occur as a result of muscle disease, such as muscular dystrophy, exposure to myotoxic agents, ischemia, and exposure to hot or cold temperatures^19^. However, focal myopathy in the SCM muscle may be rare, and neonates of uncomplicated pregnancy may rarely have events causing injury to the SCM muscle, such as exposure to hot or cold temperatures and ischemia. In addition, our data, which shows the correlation between clavicular fracture in patients with CMT and body weight at birth, suggests that mechanical injury to SCM by overstretching – as a result of difficult delivery – could cause ischemia, compartment syndrome, and/or hematoma of SCM that have been known to be related to the development of CMT. However, CMT occurs not only in subjects born through vaginal delivery, but also in those born through cesarean section. Therefore, other causes should not be excluded.

**Figure 3:**
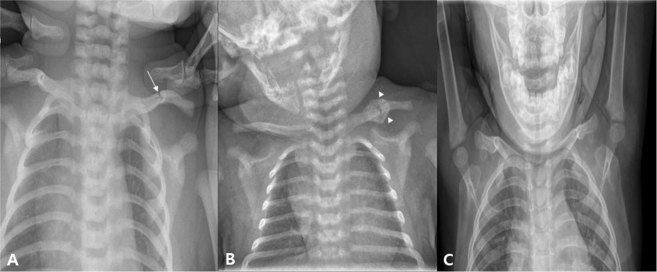
An infant who visited our hospital for left torticollis. In ultrasound examination, there was muscular fibromatosis in right SCM. (**A**) At 4 days after birth, left clavicular fracture was seen (arrow). (**B**) Follow-up plain radiograph at 21 days after birth. A callus formation was seen around fracture site of left clavicle (arrowhead). For clavicular fracture, only a conservative treatment was performed except stretching exercise and home education for right CMT. (**C**) Follow-up plain radiograph at 22 month after birth. Callus formation was disappeared, and it is difficult to find the difference between left and right clavicle. CMT; congenital muscular torticollis, SCM; sternocleidomastoid.

**Figure 4:**
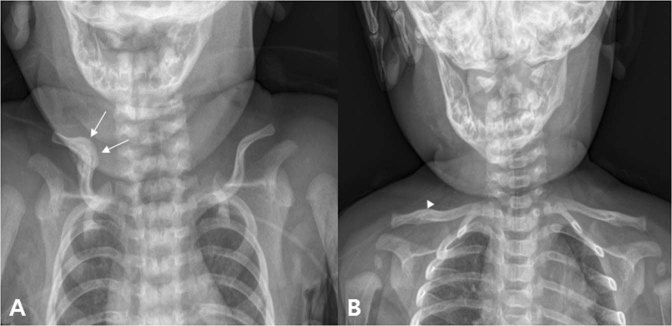
An infant who visited our hospital for left torticollis at 1 month after birth. In ultrasound examination, there was muscular fibromatosis in left SCM. (**A**) At 1 month after birth, callus formation around the fracture site of right clavicle was seen (arrow). For clavicular fracture, only a conservative treatment was performed except stretching exercise and home education for left CMT. (**B**) Follow-up plain radiograph at 4 month after birth. Callus formation was nearly disappeared, and it is difficult to find the difference between left and right clavicle (arrowhead).

The incidence of clavicular fractures in newborns ranges from 0.01 to 1.65 percent, and the incidence of clavicular fracture in patients with CMT in the study of 996 patients with CMT was 2.01%^6,20–23^. Considering the prevalence of CMT, the prevalence of clavicle fractures in CMT patients is very low in infants as a whole. However, in our study, the incidence of clavicular fracture in patients with CMT was 11.19%. This is likely due to two reasons: First, we did not perform routine x-ray examinations for clavicular fractures in all CMT children, but performed in patients with suspected clavicular fractures on physical examination, such as decreased Moro reflex, swelling, mass, tenderness, and crepitation of the affected side. However, in our study, only CMT patients who received x-rays were enrolled; the incidence of clavicular fracture in CMT patients seems to be higher than in previous studies. Second, more complicated SCM patients tend to be admitted to our hospital, as they are referred from local primary clinics due to the nature of the medical system in Korea. However, considering the relatively high incidence rate of clavicular fracture in CMT patients and the association between clavicular fracture and increased body weight at birth, a more thorough evaluation seems necessary. In this study, only a conservative treatment was performed in CMT patients with clavicular fracture, except for the incorporation of stretching exercise and home education for CMT. On follow-up plain radiography, however, most of the CMT patients with clavicular fracture showed a tendency to recover well without deformation of the clavicle (Figs 3 and 4).

There are several limitations in our study. First, the number of CMT patients with clavicular fracture included was relatively small. However, since clavicular fracture in patients with CMT is rare, this study, evaluating its risk factors, may be meaningful. In the future, further studies with a large number of patients may be necessary to better evaluate the risk factors of clavicular fracture in CMT patients. Second, selection bias may be present due to the retrospective nature of this study. As aforementioned, we did not perform radiologic evaluation on all CMT children; These may have impacted our incidence rate of clavicular fracture in patients with CMT. However, to the best of our knowledge, this is the first report on the risk factor of clavicular fracture in infants with CMT. The results of our study may be helpful in diagnosing clavicular fracture in CMT infants by informing clinicians with the relevant risk factors for clavicular fractures that may be under-diagnosed.

## Conclusions

This is the first report on the risk factors of clavicular fracture in CMT infants and its effect on prognosis. Birth weight can be a clinical predictor for clavicular fracture in CMT infants. However, presence of clavicular fracture is not associated with treatment duration. This study suggests that physiatrists need to be aware of the possibility of a clavicular fracture in CMT infants when the birth weight is greater than 3470 g. More studies and discussions are needed on whether screening test should be recommended for detecting the concurrent clavicular fracture in CMT infants.

## References

[1] Cheng JC, Au AW (1994). Infantile torticollis: a review of 624 cases. Journal of pediatric orthopedics.

[2] Do TT (2006). Congenital muscular torticollis: current concepts and review of treatment. Current opinion in pediatrics.

[3] Nilesh K, Mukherji S (2013). Congenital muscular torticollis. Annals of maxillofacial surgery.

[4] Stellwagen L, Hubbard E, Chambers C, Jones KL (2008). Torticollis, facial asymmetry and plagiocephaly in normal newborns. Archives of disease in childhood.

[5] Aarnivala HE, Valkama AM, Pirttiniemi PM (2014). Cranial shape, size and cervical motion in normal newborns. Early human development.

[6] Yim Shin-Young, Chang Kihong, Ahn Ah-Reum, Park Eun Ji, Kim Jongwoo (2018). Contralateral Involvement of Congenital Muscular Torticollis and Clavicular Fracture. American Journal of Physical Medicine & Rehabilitation.

[7] Kim MY, Kwon DR, Lee HI (2009). Therapeutic effect of microcurrent therapy in infants with congenital muscular torticollis. PM & R: the journal of injury, function, and rehabilitation.

[8] Lee SJ (2011). Comparison of clinical severity of congenital muscular torticollis based on the method of child birth. Annals of rehabilitation medicine.

[9] Hervey-Jumper SL, Justice D, Vanaman MM, Nelson VS, Yang LJ (2011). Torticollis associated with neonatal brachial plexus palsy. Pediatric neurology.

[10] Nath RK, Avila MB, Karicherla P, Somasundaram C (2011). Assessment of triangle tilt surgery in children with obstetric brachial plexus injury using the pediatric outcomes data collection instrument. The open orthopaedics journal.

[11] von Heideken J (2006). The relationship between developmental dysplasia of the hip and congenital muscular torticollis. Journal of pediatric orthopedics.

[12] Rutgers, R. A., Bilo, R. A., Nijs, H. G., Bosschaart, A. N. & van Rijn, R. R. [Fractures in full-term neonates]. *Nederlands tijdschrift voor geneeskunde* **151**, 1043; author reply 1043–1044 (2007). 17508693

[13] Yim SY (2010). The laryngeal cough reflex in congenital muscular torticollis: is it a new finding?. American journal of physical medicine & rehabilitation.

[14] Lee YT (2011). Risk factors for intrauterine constraint are associated with ultrasonographically detected severe fibrosis in early congenital muscular torticollis. Journal of pediatric surgery.

[15] Davids JR, Wenger DR, Mubarak SJ (1993). Congenital muscular torticollis: sequela of intrauterine or perinatal compartment syndrome. Journal of pediatric orthopedics.

[16] Tang S, Liu Z, Quan X, Qin J, Zhang D (1998). Sternocleidomastoid pseudotumor of infants and congenital muscular torticollis: fine-structure research. Journal of pediatric orthopedics.

[17] Hollier L, Kim J, Grayson BH, McCarthy JG (2000). Congenital muscular torticollis and the associated craniofacial changes. Plastic and reconstructive surgery.

[18] Cheng JC (2001). Clinical determinants of the outcome of manual stretching in the treatment of congenital muscular torticollis in infants. A prospective study of eight hundred and twenty-one cases. The Journal of bone and joint surgery. American volume.

[19] Seo SJ (2013). Is craniofacial asymmetry progressive in untreated congenital muscular torticollis?. Plastic and reconstructive surgery.

[20] Bhat BV, Kumar A, Oumachigui A (1994). Bone injuries during delivery. Indian journal of pediatrics.

[21] Beals RK, Sauser DD (2006). Nontraumatic disorders of the clavicle. The Journal of the American Academy of Orthopaedic Surgeons.

[22] Kaplan B (1998). Fracture of the clavicle in the newborn following normal labor and delivery. International journal of gynaecology and obstetrics: the official organ of the International Federation of Gynaecology and Obstetrics.

[23] Lurie S, Wand S, Golan A, Sadan O (2011). Risk factors for fractured clavicle in the newborn. The journal of obstetrics and gynaecology research.

